# Interphase fluorescence in situ hybridization improves the detection of malignant cells in effusions from breast cancer patients.

**DOI:** 10.1038/bjc.1997.65

**Published:** 1997

**Authors:** N. Zojer, M. Fiegl, J. Angerler, L. Müllauer, A. Gsur, S. Roka, M. Pecherstorfer, H. Huber, J. Drach

**Affiliations:** First Department of Internal Medicine, Division of Clinical Oncology, University of Vienna, Austria.

## Abstract

**Images:**


*British Journal of Cancer* (1997) **75**(3), 403–407
© 1997 Cancer Research Campaign

### Interphase fluorescence in situ hybridization improves the detection of malignant cells in effusions from breast cancer patients

**N Zojer[1], M Fiegl[1], J Angerler[1], L Müllauer[2], A Gsur[1], S Roka[1], M Pecherstorfer[3], H Huber[1] and J Drach[1]**

[1]First Department of Internal Medicine, Division of Clinical Oncology and [2]Department of Clinical Pathology, University of Vienna, Vienna, Austria; [3]First Department of Internal Medicine with Oncology, Wilhelminenspital, Vienna, Austria

**Summary** In diagnostic evaluation of effusions, difficulties are encountered when atypical reactive mesothelial cells have to be differentiated from malignant cells. We tested the impact of fluorescence in situ hybridization (FISH) to identify metastatic cells in breast cancer effusions by detection of numerical chromosomal changes. Pleural and ascitic fluid samples ($n$=57) from 41 breast cancer patients were concomitantly evaluated by routine cytology and FISH, using centromere-specific probes representing chromosomes 7, 11, 12, 17 and 18. After setting stringent cut-off levels deduced from non-malignant control effusions ($n$=9), the rates of cells with true aneuploidy were determined in each effusion sample from breast cancer patients. The occurrence of aneuploid cells, as detected by FISH and indicative of malignancy, was correlated with the cytological findings. Routine cytology revealed malignancy in 60% of effusions. Using FISH, aneuploid cell populations could be observed in 94% of cytologically positive and in 48% of cytologically negative effusions, thus reverting diagnosis to malignancy. To confirm malignancy in cases with a low frequency of aneuploid cells, two-colour FISH was additionally performed and indeed showed heterogeneous chromosomal aneuploidy within single nuclei. We conclude that FISH is a valuable tool in the diagnosis of malignancy and may serve as an adjunct to routine cytological examination, as demonstrated here for breast cancer effusions.

**Keywords:** interphase cytogenetics; breast cancer; aneuploidy; malignant effusion

Interphase cytogenetics by fluorescence in situ hybridization has gained broad use in basic research to delineate common chromosomal abnormalities in haematological malignancies and solid tumours. However, there are only few reports describing a potential clinical application of FISH with diagnostic and prognostic significance (Escudier et al, 1993; Taylor et al, 1993; Bandyk et al, 1994; Drach et al, 1995*a*; Tanner et al, 1995). In previous work from our laboratory, evidence was obtained that chromosomal abnormalities by FISH are present in cancerous specimens from all breast cancer patients studied (Fiegl et al, 1995); aneuploidy was also identified in cytologically negative effusions from breast cancer patients. This is of interest as cytological diagnosis of malignancy in effusions from cancer patients is hampered because of difficulties in differentiating malignant cells from reactive mesothelial cells (Starr and Sherman, 1991).

The aim of this study was to test the usefulness of FISH as a complementary diagnostic tool for detection of malignant cells. Thus, we (1) determined the occurrence of aneuploidy for chromosomes 7, 11, 12, 17 and 18 in effusions from breast cancer patients, (2) correlated these findings with concomitantly achieved cytological diagnosis and (3) investigated whether background non-diploidy is present in control effusions.

*Received 23 April 1996*
*Revised 17 July 1996*
*Accepted 20 August 1996*

*Correspondence to:* N Zojer, University of Vienna, Department of Internal Medicine I, Division of Clinical Oncology, Währinger Gürtel 18–20, A-1090 Vienna, Austria

### MATERIALS AND METHODS

### Clinical material

Fifty-seven consecutive effusion specimens (34 pleural and 23 ascitic) from 41 breast cancer patients and nine effusions (four pleural and five ascitic) from patients with non-malignant disease were subjected to routine diagnostic evaluation (haematoxylin–eosin, papanicolau, giemsa stains) and to FISH studies using centromeric probes for five chromosomes. All breast cancer patients (aged between 41 and 85 years, median 58 years) were considered as clinical stage IV; the lag time from first diagnosis of breast cancer to punctation of effusion ranged from 0.5 to 22 years (median 4 years). In ten cases (24%), development of effusion was the first sign of generalization. An aliquot of 50 ml of the effusion specimens was submitted to the Department of Pathology for cytological evaluation, and at least 1500 nucleated cells per slide were screened after preparation of three cytospins from each sample.

### FISH procedures

Depending on availability and cellular density by rapid staining (Diff. Quick), cells from 200–2000 ml of effusion fluid were gained by centrifugation, and, in case of macroscopic blood contamination, by gradient separation (Ficoll, $n$=7). Pelleted effusion cells were washed twice in phosphate-buffered saline (PBS), fixed in methanol–acetic acid (3:1, v/v) and stored at –80° C.

Directly fluorescence-labelled $\alpha$-satellite probes (either Spectrum-green or Spectrum-orange; Imagenetics, Framingham, MA, USA), specific for the centromeric regions of human chromosomes 7, 11, 12, 17 and 18, were applied, following the protocol described by Drach et al (1995*b*). In 13 effusions, chromosomes 11 and 17 were

**Table 1** FISH results of pleural and peritoneal fluid samples from patients with non-malignant disease (n=9)

| | Signal number per nucleus | | | | | |
|---|---|---|---|---|---|---|
| Chromosome | 1 | 2 | 3 | 4 | 5 | 6 |
| 7 | 5.11 ± 1.08 | 94.41 ± 1.11 | 0.23 ± 0.11 | 0.23 ± 0.37 | 0 | 0.02 ± 0.05 |
| 11 | 5.96 ± 1.88 | 93.49 ± 1.60 | 0.20 ± 0.20 | 0.33 ± 0.52 | 0.02 ± 0.05 | 0 |
| 12 | 5.30 ± 1.39 | 93.85 ± 0.92 | 0.52 ± 0.38 | 0.33 ± 0.47 | 0 | 0 |
| 17 | 7.59 ± 2.28 | 91.73 ± 1.88 | 0.34 ± 0.29 | 0.34 ± 0.66 | 0 | 0 |
| 18 | 7.45 ± 1.35 | 92.00 ± 0.98 | 0.31 ± 0.31 | 0.24 ± 0.44 | 0 | 0 |

Data are given as mean percentage ± s.d. of centromeric signal numbers. At least 1000 nuclei were analysed per sample and chromosome. Mean percentages + 3 s.d. were calculated for definition of the cut-off levels.

 © Cancer Research Campaign 1997

**Table 2** Classification of 57 effusions from breast cancer patients by cytology and FISH

| No. | Site[b] | C[c] | FISH[c] | Aneuploidy rate[a] for chromosome 7 | 11 | 12 | 17 | 18 |
|---|---|---|---|---|---|---|---|---|
| 1 | P | Neg | Neg | 0 | 0 | 0 | 0 | 0 |
| 2 | P | Neg | Neg | 0 | 0 | 0 | 0 | 0 |
| 3 | P | Neg | Neg | 0 | 0 | 0 | 0 | 0 |
| 4 | P[h] | Neg | Neg | 0 | 0 | 0 | 0 | 0 |
| 5 | P | Neg | Neg | 0 | 0 | 0 | 0 | 0 |
| 6 | P | Neg | Neg | 0 | 0 | 0 | 0 | 0 |
| 7 | A | Neg | Neg[q] | 0.6 | 0 | 0.2 | 0.5 | 2.8 |
| 8 | A | Neg | Neg | 0 | 0 | 0 | 0 | 0 |
| 9 | A[i] | Neg | Neg | 0 | 0 | 0 | 0 | 0 |
| 10 | A[i] | Neg | Neg | 0 | 0 | 0 | 0 | 0 |
| 11 | A[j] | Neg | Neg | 0 | 0 | 0 | 0 | 0 |
| 12 | A[j] | Neg | Neg | 0 | 0 | 0 | 0 | 0 |
| 13 | P | Pos | Pos | 0 | 11.3 | 0 | 8 | 0 |
| 14 | P | Pos | Pos | 24.9 | 24.1 | 22.4 | 6.5 | 0 |
| 15 | P | Pos | Pos | 5.6 | 53.5 | 12.5 | 44.8 | 6.1 |
| 16 | P | Pos | Pos | 3.1 | 51.1 | 13.4 | 90 | 0 |
| 17 | P | Pos | Pos | 0.4 | 28.6 | 5 | 11.5 | 0 |
| 18 | P | Pos | Pos | 0 | 17.1 | 0.2 | 2.8 | 0 |
| 19 | P | Pos | Pos | 0.6 | 11.3 | 0 | 10 | 0 |
| 20 | P | Pos | Pos | 15.1 | 80.1 | 14.4 | 86 | 6.3 |
| 21 | P[h] | Pos | Pos[p] | 2.1 | 0 | 1.4 | 0 | 0.7 |
| 22 | P | Pos | Pos[p] | 0 | 0 | 0 | 0 | 0.3 |
| 23 | P | Pos | Pos | 0 | 5.6 | 2.1 | 31.3 | 9.6 |
| 24 | P | Pos | Pos[p] | 2.9 | 0 | 0 | 3.8 | 4.6 |
| 25 | P[d] | Pos | Pos | 7.4 | 54.4 | 0 | 51.3 | 9.9 |
| 26 | P | Pos | Pos | 26.7 | 8.7 | 65.9 | 16.5 | 52.4 |
| 27 | P | Pos | Pos | 11.5 | 41.5 | 2.3 | 33.2 | 71.5 |
| 28 | P | Pos | Pos | 62.3 | 45.5 | 6.7 | 54.5 | 74.4 |
| 29 | P[k] | Pos | Pos | 24.3 | 11.9 | 47.4 | 33.1 | 0 |
| 30 | P[k] | Pos | Pos | 1.9 | 0 | 5.4 | 4.7 | 6.6[n] |
| 31 | P | Pos | Pos | 57.1 | 3.2[n] | 39.5 | 17.5 | 40.4 |
| 32 | P[l] | Pos | Pos | 6.5 | 0 | 4 | 0 | 0 |
| 33 | P[l] | Pos | Pos | 4.3 | 5.3 | 3 | 1.9 | 7.5 |
| 34 | P[l] | Pos | Pos[p] | 1 | 2.8 | 0 | 1 | 1.5 |
| 35 | A | Pos | Pos | 0 | 13.6 | 5.9 | 4.4 | 0 |
| 36 | A | Pos | Pos | 1.2 | 2.2 | 1.2 | 79.1[n] | 83[n] |
| 37 | A[f] | Pos | Pos | 31.1 | 77.3 | 39.1 | 2.9 | 0.8 |
| 38 | A[g] | Pos | Pos | 9.9 | 11 | 1.1 | 1.8 | 3.1 |
| 39 | A[g] | Pos | Pos | 0.8 | 21 | 2.1 | 3.9 | 0.5 |
| 40 | A[g] | Pos | Pos | 1.2 | 15.1 | 1.1 | 8.9 | 0 |
| 41 | A[g] | Pos | Pos | 3.8 | 34.6 | 0 | 7.4 | 0 |
| 42 | A | Pos | Pos | 0 | 1.5 | 3.3 | 6.9 | 16.2 |
| 43 | A[d] | Pos | Pos | 0 | 6.2 | 0 | 3.3 | 0 |
| 44 | A | Pos | Pos | 62.8 | 72.1 | 65.4 | 68.4 | 58.7 |
| 45 | P | Pos | Neg | 0 | 0 | 0 | 0 | 0 |
| 46 | P | Pos | Neg[q] | 0 | 0 | 0 | 0 | 0.5 |
| 47 | P | Neg | Pos | 0 | 5.8 | 0.7 | 1.8 | 0.8 |
| 48 | P[e] | Neg | Pos | 0 | 3.8 | 0.6 | 80[n] | 0 |
| 49 | P[m] | Neg | Pos | 12 | 16.4 | 17.6 | 0 | 33.5 |
| 50 | P[m] | Neg | Pos | 11.6 | 4.4 | 9.4 | 3.9 | 9.9 |
| 51 | A[e] | Neg | Pos | 0 | 9.1 | 3.8 | 67.1[n] | 0 |
| 52 | A[e] | Neg | Pos | 5.2 | 1.8 | 0 | 69.2[n] | 1.4 |
| 53 | A[f] | Neg | Pos | 64.1 | 72.8 | 60.2 | 5.8 | 1 |
| 54 | A[f] | Neg | Pos | 56.8 | 72.9 | 53.5 | 0.8 | 0 |
| 55 | A | Neg | Pos | 0.5 | 6.9 | 1 | 1 | 0 |
| 56 | A | Neg | Pos[p] | 0 | 0.5 | 0.3 | 0 | 0.6 |
| 57 | A[i] | Neg | Pos[p] | 0.5 | 0 | 1.2 | 0 | 0 |

[a]Aneuploidy rate is the sum of percentages of monosomy and ≥ trisomy above cut-off. [b]P, pleural effusion; A, ascitic effusion. [c]Cytological and FISH classification of effusion. Neg, negative; Pos, positive.[e–m] Effusions from the same patient. [n]Predominant[d–m] monosomy. [p]Results confirmed by two-colour FISH. [q]Classification according to two-colour FISH; results of single-hybridization experiments could not be confirmed.

targeted with biotin- and digoxigenin-labelled probes (Fiegl et al, 1995), which were obtained from Oncor (Gaithersburg, MD, USA), and detection was accomplished using FITC-conjugated avidin and anti-digoxigenin rhodamine respectively. In agreement with our previous experience, the confidence intervals for controls were identical for both types of probes (Escudier et al, 1993).

For two-colour studies, DNA probes representing two chromosomes and labelled with different fluorochromes were combined. In control effusions, chromosomes 11 and 18 were targeted for two-colour FISH experiments; in effusions from breast cancer patients, centromeric probes were chosen depending on the results of single hybridization experiments.

### Analysis by fluorescence microscopy

Fluorescence signals of at least 1000 nuclei from control effusions and 200–800 nuclei from breast cancer effusions were scored, with high-number cell counting in samples with low frequency of aneusomic cells (Kibbelaar et al, 1993). An Olympus AH-3 microscope with a 100 × planar objective was used for signal analysis, and the stringent criteria proposed by Hopman et al (1988) were applied. The portion of zero-spot cells (inversely correlated with hybridization efficiacy) was below 1% in all control and breast cancer effusions.

All cells in a field were scored except for granulocytes; lymphocytes were not omitted from evaluation as nuclear shape of lymphoid cells may resemble that of breast cancer cells (Johnston, 1985). Signal analysis was done without knowledge of cytological results.

### Definition of aneuploidy rate

For an unequivocal definition of true aneuploidy in breast cancer effusions, mean percentages + 3 s.d. of control cells with non-disomic signal numbers were set as cut-off levels (Bentz et al, 1993; Drach et al, 1995*a*; Fiegl et al, 1995). Percentages of monosomic, trisomic and up to hexasomic cells in breast cancer effusions were thus corrected for background aneuploidy, as derived from control effusions (Table 1). Cells with more than six signals were considered as unambiguously aneuploid. To quantitate truly aneuploid cells in breast cancer effusions, aneuploidy rate was calculated as the sum of percentages of monosomy and ≥ trisomy above the cut-off levels.

### RESULTS

### Observation of non-diploid cells in control effusions

By means of extensive cell counting, rare aneusomic cells were found in control effusions from nine patients with non-cancerous diseases (Table 1). Percentages of trisomic cells were in accordance with previous observations in normal lymphocytes (Fiegl et al, 1995; Herrington et al, 1995). However, a significant proportion of mesothelial cells, distinguished by size and shape, exhibited tetrasomy for all chromosomes examined; in three effusions also pentasomy and/or hexasomy was observed. As detected by two-colour FISH, there was equality in copy numbers for chromosome 11 and 18 in these mesothelial cells, indicative of polyploidy rather than numerical changes of single chromosomes (not shown), being in accordance with previous reports on polyploidy in mesothelial cells (Bousfield et al, 1984; Biesterfeld et al, 1994).

Cut-off levels were calculated as outlined in the methods section.

© Cancer Research Campaign 1997

### Aneuploidy for chromosomes 7, 11, 12, 17 and 18 in cytologically malignant breast cancer effusions

Of 57 consecutive effusions, 34 (60%) were diagnosed as malignant following routine cytological procedures, frequency being in line with previous reports (Leuallen and Carr, 1955; Banerjee et al, 1994). Pleural effusions were cytologically positive in 71%, whereas malignancy could be found in 43% of ascitic effusions.

In order to define the frequency of aneuploidy in cytologically positive effusions, FISH using centromeric probes representing chromosomes 7, 11, 12, 17 and 18 was performed. Of the 34 cytologically positive effusions, 28 contained aneusomic cells in more than 5% above the defined cut-off and five effusions in a percentage below 5%. In one effusion, no aneuploidy was detected by FISH using these five centromeric probes, as shown in Table 2.

The extent of aneuploidy in all effusions is listed in Table 2, using an index based on the signal counting results (aneuploidy rate). To confirm diagnosis of malignancy in the five effusions with aneuploidy rates below 5%, two-colour FISH was performed. Heterogeneous chromosomal abnormalities within single cells were indeed demonstrated in four cases (Figure 1). One of the five effusion specimens was classified as being non-malignant according to two-colour FISH. Thus, 32 of the 34 cytologically positive effusions (94%) fulfilled our criteria of malignancy, as detected by FISH.

The counting results concerning chromosomes 11 and 17 for 13 effusions were detailed in our previous report (Fiegl et al, 1995). In the extended series presented in this study, all FISH-positive cases showed predominantly gain for chromosomes 7, 11 and 12; however, predominance of monosomy 17 in four cases (11% of effusions with aneuploidy for chromosome 17), of monosomy 18 in two cases (8% of effusions positive for chromosome 18) and of monosomy 11 in one case was observed. Furthermore, tumour cell heterogeneity for all five chromosomes was detected, mostly with a wide range of centromeric signal numbers.

The constant presence of aneuploidy in cytologically positive effusions suggests that this finding can be used as an indicator of malignancy in diagnostic procedures.

### FISH detects aneuploid cells in cytologically negative effusions

Twenty-three cytologically negative effusions were examined by FISH for the potential occurrence of malignancy, undetectable by cytological criteria only. Interphase cytogenetics, including two-colour FISH, revealed true aneuploidy in 11 of these effusion specimens (48%). Combined aneuploidy rates for all five chromosomes ranged from 0.6% to 80% (median 11.6%).

Combination of centromeric probes for chromosomes 7 and 11 represented the most successful combination of two probes as 42 out of the 43 FISH-positive effusions were detected. In 12 effusions, neither FISH nor cytology yielded positivity, giving evidence of a reactive genesis of effusions, e.g. portal hypertension owing to metastasis to the liver.

Analysing results according to effusion site, FISH improved diagnostic sensitivity in the case of ascitic effusions (74% positivity *vs* 43% positivity by routine cytological evaluation; $P < 0.05$, using the $\chi^2$-test). Nevertheless, aneuploid cell populations could also be demonstrated in four cytologically negative pleural effusions (76% positivity *vs* 71% cytological positivity; $P$=NS). When considering all positive effusions (by FISH and/or cytology; $n$=45), malignancy could be detected in 96% ($n$=43) by FISH alone, whereas positivity was diagnosed in only 76% ($n$=34) by routine cytological criteria ($P$<0.01).

### DISCUSSION

This is the first systematic study investigating interphase cytogenetics as a tool in the evaluation of breast cancer effusions for malignancy. Cancerous cells in effusions from breast cancer patients are detectable in about 50% using routine cytology (Banerjee et al, 1994; Leuallen et al, 1955). Difficulties are encountered when atypical reactive mesothelial cells have to be differentiated from malignant cells (Starr et al, 1991). Thus, considerable effort has been undertaken to improve tumour cell detection in effusions from cancer patients by means of immunocytochemistry, DNA cytometry and metaphase cytogenetics (Bousfield et al, 1985; Loy et al, 1990; Gioanni et al, 1991; Osinaga et al, 1992; Athanassiadou et al, 1994; Joseph et al, 1995). Because of problems concerning sensitivity and/or specificity and technical limitations, these methods have not gained broad clinical significance in diagnostic evaluation of effusions. However, as the Ber-EP4 antibody distinguishes between epithelial and mesothelial cells (Latza et al, 1990; De-Angelis et al, 1992; Illingworth et al, 1994), it would be interesting to compare FISH analysis with an immuno-cytochemical approach using the Ber-EP4 antibody, either alone or as part of a panel of antibodies.

In breast cancer, no specific genetic alteration is present which would allow screening for an isolated chromosomal change. However, disease progression leads to accumulation of chromosomal alterations, resulting in complex karyotypes (Heim et al, 1988; Dutrillaux et al, 1991; Trent et al, 1993), thus enabling interphase cytogenetics to be used in tumour cell detection when a panel of centromeric probes is applied. Indeed, aneuploid cell populations could be demonstrated with FISH in 43 of the 57 effusions in our series (75%), and, more interestingly, in 11 of the 23 (48%) effusions with a negative cytological diagnosis. FISH results of cells separated over a Ficoll–Hypaque gradient (seven effusions) reflected the results of the whole series, thus excluding a major influence of cell enrichment by Ficoll on the data in this subset of effusions.

In two cytologically positive effusions, aneuploid cells could not be detected by interphase cytogenetics using five centromeric probes. Use of a larger panel of FISH probes for screening might reveal chromosomal changes in these cases too, as breast cancer is a genetically extremely heterogeneous disease with aneuploidy detected in the vast majority of cases (Beerman et al, 1991; Teixeira et al, 1994; Fiegl et al, 1995; Pandis et al, 1995). On the other hand, enrichment of cells expressing epithelial markers by flow cytometry and consecutive FISH analysis might improve the detection limit of interphase cytogenetics in the case of rare breast cancer cells.

For FISH studies, adequate control material is crucial as clonal chromosomal changes may physiologically occur in different non-neoplastic cells (Richard et al, 1993; Casalone et al, 1995), and FISH-inherent artifacts may also contribute to background non-diploidy. Our results of cells with monosomic and trisomic signals in control effusions are in good agreement with results obtained by other investigators (Eastmond et al, 1995; Herrington et al, 1995). It is generally believed that overlapping signals are the main reason for monosomy in control cells, whereas non-specific binding and signal-splitting is thought to be responsible for the trisomy observed in these cases. We also observed rare tetrasomic cells in our control effusions, propably due to the occurrence of polyploid mesothelial cells. By setting stringent cut-off levels,

© Cancer Research Campaign 1997

background non-diploidy was differentiated from aneuploidy, clearly indicating malignancy.

In the effusions with low but significant aneuploidy rates (e.g. effusion sample 22), the percentage of counted nuclei with three or more signals was always more than 1%, which is considered as the lower detection limit of FISH analysis (Kibbelaar et al, 1993). To validate specificity, all effusions with an aneuploidy rate below 5% were reviewed using two-colour FISH. Hereby, malignancy in six samples could be confirmed, whereas the finding of polyploid cells only (similar to controls) in two of the reviewed effusions suggested a rather reactive origin.

Development of effusion occurs in 50% of breast cancer patients (Raju and Kardinal, 1981). In this study, FISH was shown to be suitable for detection of malignancy in effusions and to improve sensitivity of diagnosis at high specificity. This may be of great value, especially in the 20% of cases in which effusion is the only sign of recurrence (Raju and Kardinal, 1981; as shown in our series also) and other causes of fluid generation need to be excluded. As anti-tumour therapy requires reliable diagnosis of malignancy, (micro)-metastasis detection by FISH may lead to rapid initiation of adequate treatment.

### ACKNOWLEDGEMENT

This work was kindly supported by Glaxo Wellcome Pharma Gmbh.

### REFERENCES

Athanassiadou P, Athanassiades P, Lazaris D, Kyrkou K, Petrakakou E and Aravantinos D (1994) Immunocytochemical differentiation of reactive mesothelial cells and adenocarcinoma cells in serous effusions with the use of carcinoembryonic antigen and fibronectin. *Acta Cytol* **38**: 718–722

Bandyk MG, Zhao L, Troncoso P, Pisters LL, Palmer JL, Von Eschenbach AC, Chung LWK and Liang JC (1994) Trisomy 7: a potential cytogenetic marker of human prostate cancer progression. *Genes Chrom Cancer* **9**: 19–27

Banerjee AK, Willetts I, Robertson JFR and Blamey RW (1994) Pleural effusion in breast cancer: a review of the Nottingham experience. *Eur J Surg Oncol* **20**: 33–36

Beerman H, Smit VTHBM, Kluin PM, Bonsing BA, Hermans J and Cornelisse CJ (1991) Flow cytometric analysis of DNA stemline heterogeneity in primary and metastatic breast cancer. *Cytometry* **12**: 147–154

Bentz M, Schröder M, Herz M, Stilgenbauer S, Lichter P and Döhner H (1993) Detection of trisomy 8 on blood smears using fluorescence in situ hybridization. *Leukemia* **7**: 752–757

Biesterfeld S, Gerres K, Fischer-Wein G and Böcking A (1994) Polyploidy in non-neoplastic tissues. *J Clin Pathol* **47**: 38–42

Bousfield LR, Greenberg ML and Pacey F (1985) Cytogenetic diagnosis of cancer from body fluids. *Acta Cytol* **29**: 768–774

Casalone R, Minelli E, Righi R, Granata P, Meroni E, Caruso V, Mazzola D, Salvadore M, Pozzi E and Bono AV (1995) Clonal chromosome changes in non-neoplastic ureters. *Cancer Genet Cytogenet* **83**: 28–31

De-Angelis M, Buley ID, Heryet A and Gray W (1992) Immunocytochemical staining of serous effusions with the monoclonal antibody Ber-EP4. *Cytopathology* **3**: 111–117

Drach J, Angerler J, Schuster J, Rothermundt C, Thalhammer R, Haas OA, Jäger U, Fiegl M, Geissler K, Ludwig H and Huber H (1995*a*) Interphase fluorescence in situ hybridization identifies chromosomal abnormalities in plasma cells from patients with monoclonal gammopathy of undetermined significance. *Blood* **86**: 3915–3921

Drach J, Schuster J, Nowotny H, Angerler J, Rosenthal F, Fiegl M, Rothermundt C, Gsur A, Jäger U, Heinz R, Lechner K, Ludwig H and Huber H (1995*b*) Multiple myeloma: high incidence of chromosomal aneuploidy as detected by interphase fluorescence in situ hybridization. *Cancer Res* **55**: 3854–3859

Dutrillaux B, Gerbault-Seureau M, Remvikos Y, Zafrani B and Prieur M (1991) Breast cancer genetic evolution: I. Data from cytogenetics and DNA content. *Breast Cancer Res Treat* **19**: 245–255

Eastmond DA, Schuler M and Rupa DS (1995) Advantages and limitations of using fluorescence in situ hybridization for the detection of aneuploidy in interphase human cells. *Mutation Res* **348**: 153–162

Escudier SM, Pereira-Leahy JM, Drach JW, Weier HU, Goodacre AM, Cork MA, Trujillo JM, Keating MJ and Andreef M (1993) Fluorescent in situ hybridization and cytogenetic studies of trisomy 12 in chronic lymphocytic leukemia. *Blood* **81**: 2702–2707

Fiegl M, Tueni C, Schenk T, Jakesz R, Gnant M, Reiner A, Rudas M, Pirc-Danoewinata H, Marosi C, Huber H and Drach J (1995) Interphase cytogenetics reveals a high incidence of aneuploidy and intratumour heterogeneity in breast cancer. *Br J Cancer* **72**: 51–55

Gioanni J, Caldani C, Zanghellini E, Mazeau C, Duplay H, Ferrua B and Schneider M (1991) A new epithelial membrane antigen (calam 27) as a marker of carcinoma in serous effusions. *Acta Cytol* **35**: 315–319

Herrington CS, Cooper K and McGee JO'D (1995) Interphase cytogenetics: analysis of numerical chromosome aberrations in isolated cells. *J Pathol* **175**: 283–295

Heim S, Mandahl N and Mitelman F (1988) Genetic convergence and divergence in tumor progression. *Cancer Res* **48**: 5911–5916

Hopman AHN, Ramaekers FCS, Raap AK, Boek JLM, Devilee P, Van der Ploog M and Vooijs GP (1988) In situ hybridization as a tool to study numeric chromosome aberrations in solid bladder tumors. *Histochemistry* **89:** 307–316

Illingworth AL, Young JA and Johnson GD (1994) Immunofluorescent staining of metastatic carcinoma cells in serous fluid with carcinoembryonic antibody, epithelial membrane antibody, AUA-1 and Ber-EP4. *Cytopathology* **5**: 270–281

Kibbelaar RE, Kok F, Dreef EJ, Kleiverda JK, Cornelisse CJ, Raap AK and Kluin PM (1993) Statistical methods in interphase cytogenetics: an experimental approach. *Cytometry* **14**: 716–724

Latza U, Niedobitek G, Schwarting R, Nekarda H and Stein H (1990) Ber-Ep4: new monoclonal antibody which distinguishes epithelia from mesothelia. *J Clin Pathol* **43**: 213–219

Leuallen EC and Carr DT (1955) Pleural effusion. A statistical study of 436 patients. *N Engl J Med* **252**: 79–83

Johnston WW (1985) The malignant pleural effusion. A review of cytopathologic diagnosis of 584 specimens from 472 consecutive patients. *Cancer* **56**: 905–909

Joseph MG, Banerjee D, Harris P, Gibson S and McFadden RG (1995) Multiparameter flow cytometric DNA analysis of effusions: a prospective study of 36 cases compared with routine cytology and immunohistochemistry. *Modern Pathol* **8**: 686–693

Loy TS, Diaz-Arias AA and Bickel JT (1990) Value of BCA-225 in the cytologic diagnosis of malignant effusions: an immunocytochemical study of 197 cases. *Modern Pathol* **3**: 294–297

Osinaga E, Pancino G, Beuzelin M, Babino A, Rodriguez D, Robello C, Tiscornia A, Phillips E, Bourguignat A and Roseto A (1992) Detection of a soluble antigen defined by monoclonal antibody $83D_4$ in serous effusions associated with breast carcinoma. *Cancer* **69**: 1745–1749

Pandis N, Jin Y, Gorunova L, Petersson C, Bardi G, Idvall I, Johansson B, Ingvar C, Mandahl N, Mitelman F and Heim S (1995) Chromosome analysis of 97 primary breast carcinomas: identification of eight karyotypic subgroups. *Genes Chrom Cancer* **12**: 173–185

Raju RN and Kardinal CG (1981) Pleural effusion in breast carcinoma: analysis of 122 cases. *Cancer* **48**: 2524–2527

Richard F, Aurias A, Couturier J, Dutrillaux AM, Flüry-Hérard A, Gerbault-Seureau M, Hoffschir F, Lamoliatte E, Lefrançois D, Lombard M, Muleris M, Prieur M, Ricoul M, Sabatier L, Viegas-Péquignot E, Volobouev V and Dutrillaux B (1993) Aneuploidy in human lymphocytes: an extensive study of eight individuals of various ages. *Mutation Res* **295**: 71–80

Starr RL and Sherman ME (1991) The value of multiple preparations in the diagnosis of malignant pleural effusions. A cost-benefit analysis. *Acta Cytol* **35**: 533–537

Tanner MM, Tirkkonen M, Kallioniemi A, Holli K, Collins C, Kowbel D, Gray JW, Kallioniemi O-P and Isola J (1995) Amplification of chromosomal region 20q13 in invasive breast cancer: prognostic implications. *Clin Cancer Res* **1**: 1455–1461

Taylor C, Patel K, Jones T, Kiely F, De-Stavola BL and Sheer D (1993) Diagnosis of Ewing's sarcoma and peripheral neuroectodermal tumour based on the detection of t(11;22) using fluorescence in situ hybridisation. *Br J Cancer* **67**: 128–133

Teixeira MR, Pandis N, Bardi G, Andersen JA, Mandahl N, Mitelman F and Heim S (1994) Cytogenetic analysis of multifocal breast carcinomas: detection of karyotypically unrelated clones as well as clonal similarities between tumour foci. *Br J Cancer* **70**: 922–927

Trent J, Yang JM, Emerson J, Dalton W, McGee D, Massey K, Thompson F and Villar H (1993) Clonal chromosome abnormalities in human breast carcinomas. II. Thirty-four cases with metastatic disease. *Genes Chrom Cancer* **7**: 194–203

© Cancer Research Campaign 1997


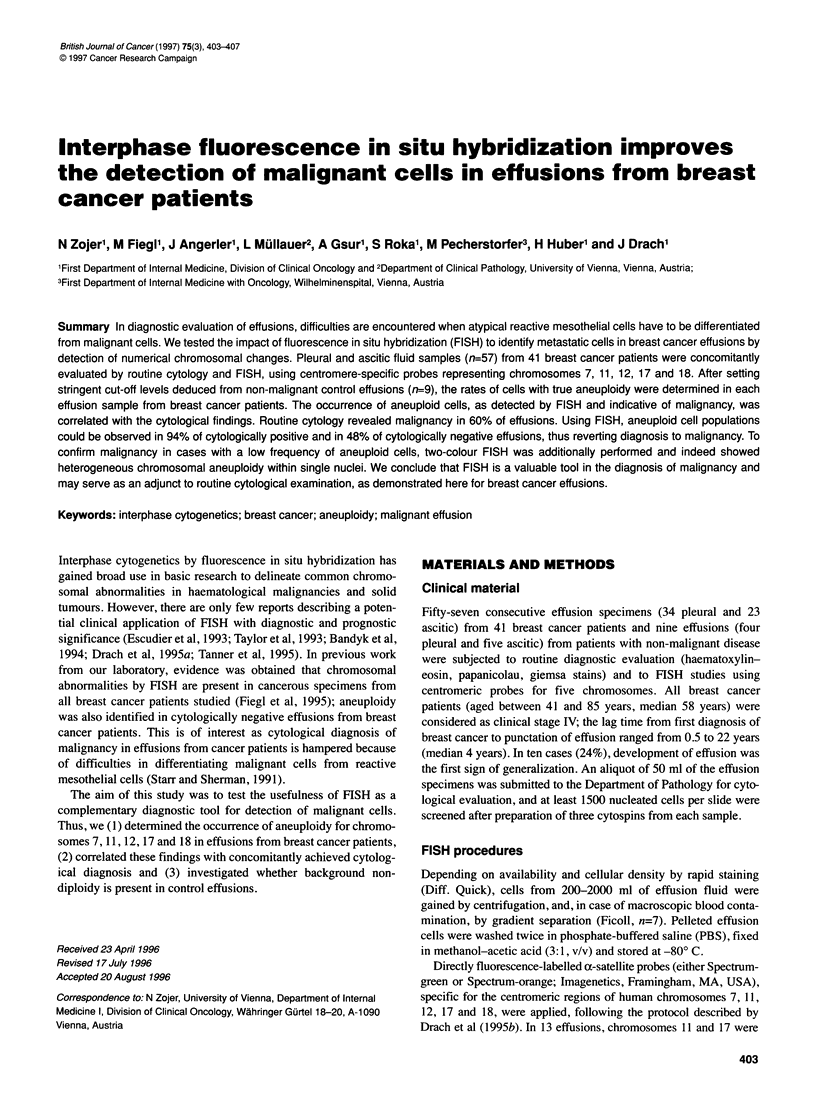

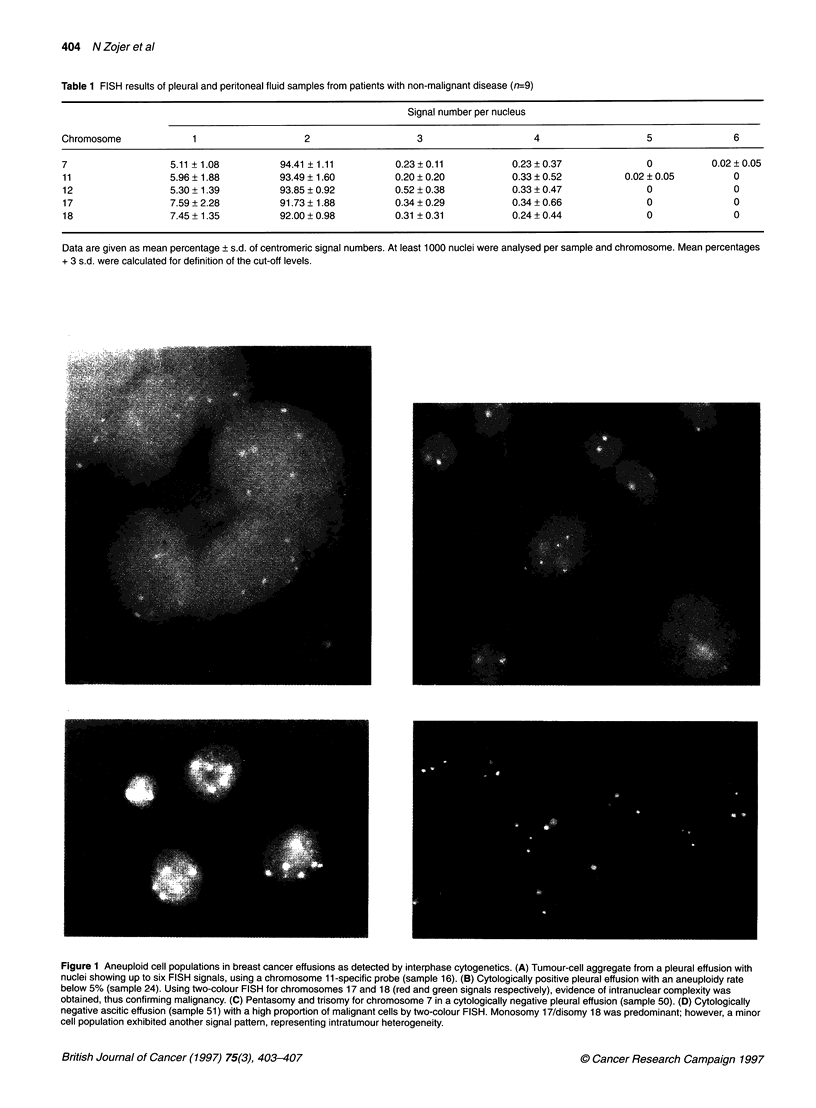

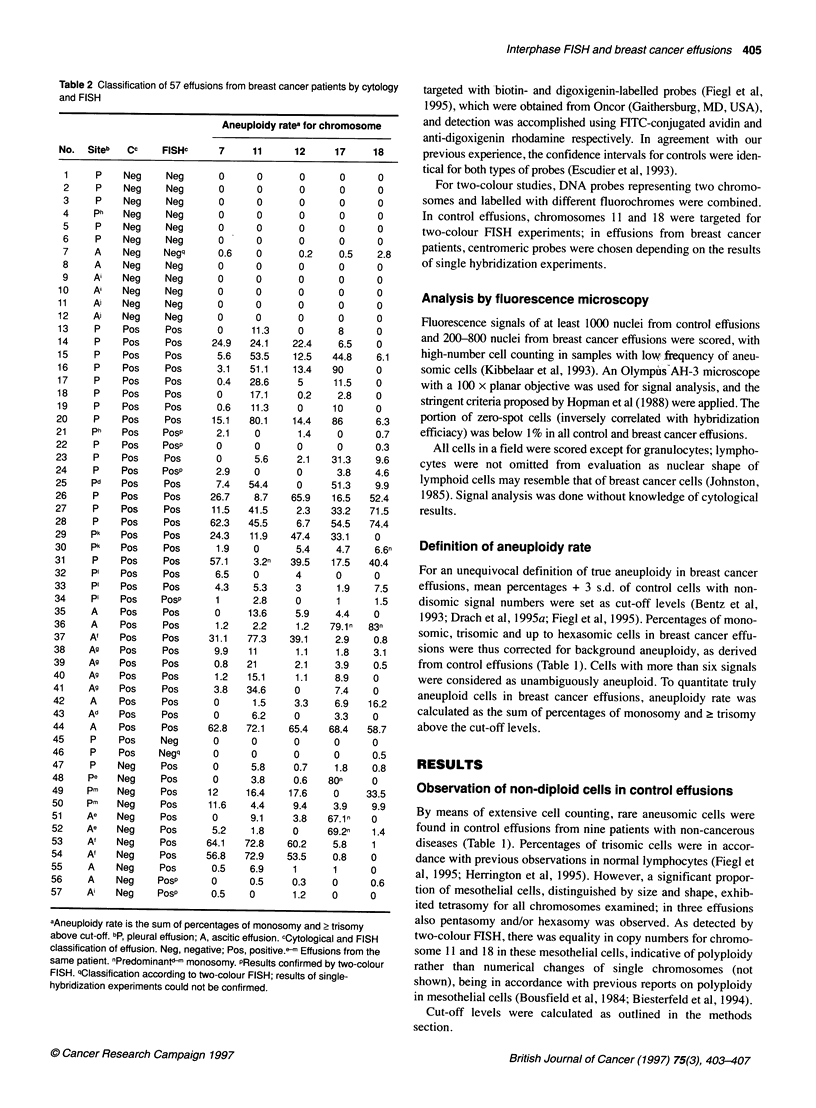

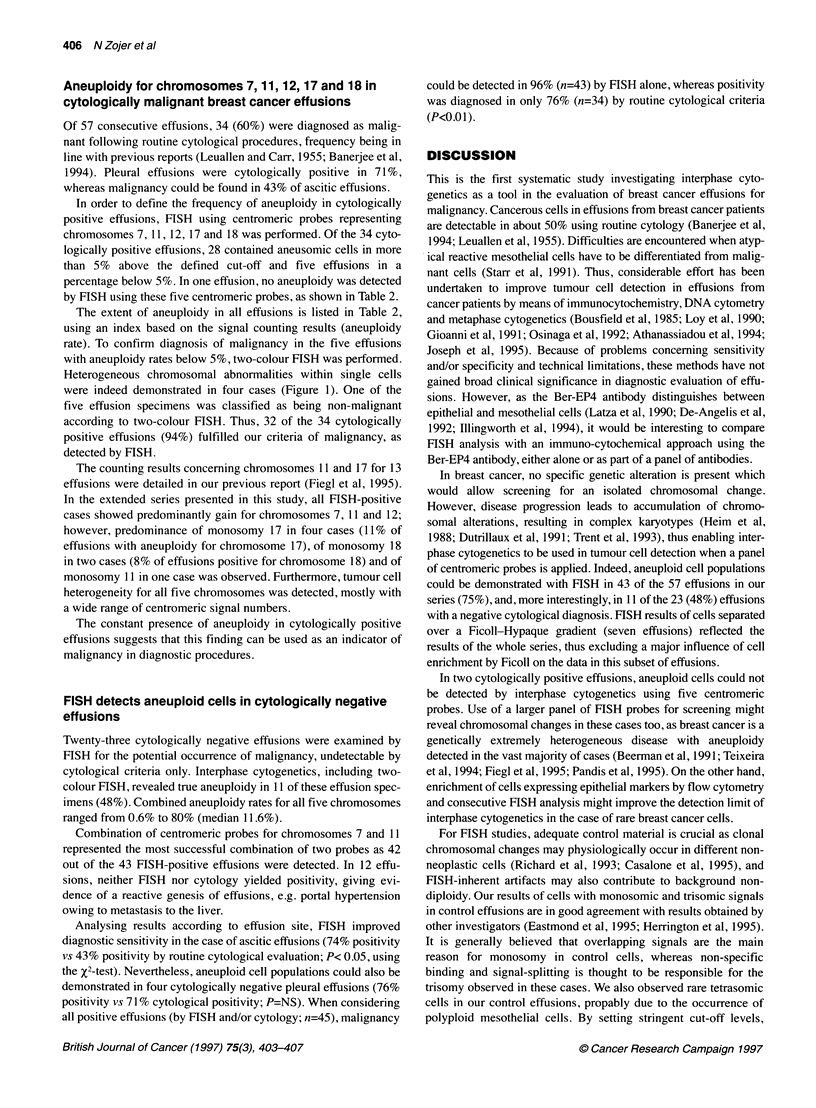

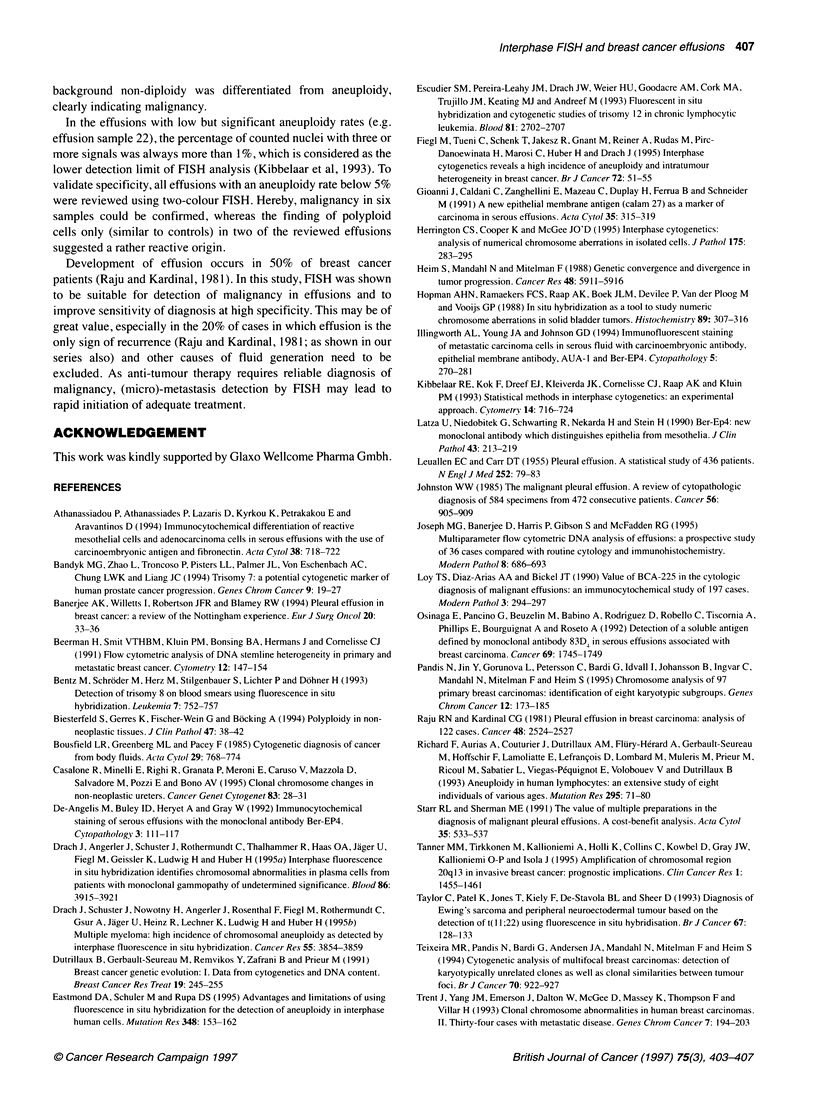


## References

[OCR_00562] Athanassiadou P., Athanassiades P., Lazaris D., Kyrkou K., Petrakakou E., Aravantinos D. (1994). Immunocytochemical differentiation of reactive mesothelial cells and adenocarcinoma cells in serous effusions with the use of carcinoembryonic antigen and fibronectin.. Acta Cytol.

[OCR_00569] Bandyk M. G., Zhao L., Troncoso P., Pisters L. L., Palmer J. L., von Eschenbach A. C., Chung L. W., Liang J. C. (1994). Trisomy 7: a potential cytogenetic marker of human prostate cancer progression.. Genes Chromosomes Cancer.

[OCR_00574] Banerjee A. K., Willetts I., Robertson J. F., Blamey R. W. (1994). Pleural effusion in breast cancer: a review of the Nottingham experience.. Eur J Surg Oncol.

[OCR_00579] Beerman H., Smit V. T., Kluin P. M., Bonsing B. A., Hermans J., Cornelisse C. J. (1991). Flow cytometric analysis of DNA stemline heterogeneity in primary and metastatic breast cancer.. Cytometry.

[OCR_00584] Bentz M., Schröder M., Herz M., Stilgenbauer S., Lichter P., Döhner H. (1993). Detection of trisomy 8 on blood smears using fluorescence in situ hybridization.. Leukemia.

[OCR_00589] Biesterfeld S., Gerres K., Fischer-Wein G., Böcking A. (1994). Polyploidy in non-neoplastic tissues.. J Clin Pathol.

[OCR_00593] Bousfield L. R., Greenberg M. L., Pacey F. (1985). Cytogenetic diagnosis of cancer from body fluids.. Acta Cytol.

[OCR_00597] Casalone R., Minelli E., Righi R., Granata P., Meroni E., Caruso V., Mazzola D., Salvadore M., Pozzi E., Bono A. V. (1995). Clonal chromosome changes in non-neoplastic ureters.. Cancer Genet Cytogenet.

[OCR_00602] De Angelis M., Buley I. D., Heryet A., Gray W. (1992). Immunocytochemical staining of serous effusions with the monoclonal antibody Ber-EP4.. Cytopathology.

[OCR_00615] Drach J., Schuster J., Nowotny H., Angerler J., Rosenthal F., Fiegl M., Rothermundt C., Gsur A., Jäger U., Heinz R. (1995). Multiple myeloma: high incidence of chromosomal aneuploidy as detected by interphase fluorescence in situ hybridization.. Cancer Res.

[OCR_00627] Eastmond D. A., Schuler M., Rupa D. S. (1995). Advantages and limitations of using fluorescence in situ hybridization for the detection of aneuploidy in interphase human cells.. Mutat Res.

[OCR_00632] Escudier S. M., Pereira-Leahy J. M., Drach J. W., Weier H. U., Goodacre A. M., Cork M. A., Trujillo J. M., Keating M. J., Andreeff M. (1993). Fluorescent in situ hybridization and cytogenetic studies of trisomy 12 in chronic lymphocytic leukemia.. Blood.

[OCR_00641] Fiegl M., Tueni C., Schenk T., Jakesz R., Gnant M., Reiner A., Rudas M., Pirc-Danoewinata H., Marosi C., Huber H. (1995). Interphase cytogenetics reveals a high incidence of aneuploidy and intra-tumour heterogeneity in breast cancer.. Br J Cancer.

[OCR_00646] Gioanni J., Caldani C., Zanghellini E., Mazeau C., Duplay H., Ferrua B., Schneider M. (1991). A new epithelial membrane antigen (Calam 27) as a marker of carcinoma in serous effusions.. Acta Cytol.

[OCR_00656] Heim S., Mandahl N., Mitelman F. (1988). Genetic convergence and divergence in tumor progression.. Cancer Res.

[OCR_00651] Herrington C. S., Cooper K., McGee J. O. (1995). Interphase cytogenetics: analysis of numerical chromosome aberrations in isolated cells.. J Pathol.

[OCR_00660] Hopman A. H., Ramaekers F. C., Raap A. K., Beck J. L., Devilee P., van der Ploeg M., Vooijs G. P. (1988). In situ hybridization as a tool to study numerical chromosome aberrations in solid bladder tumors.. Histochemistry.

[OCR_00665] Illingworth A. L., Young J. A., Johnson G. D. (1994). Immunofluorescent staining of metastatic carcinoma cells in serious fluid with carcinoembryonic antibody, epithelial membrane antibody, AUA-1 and Ber-EP4.. Cytopathology.

[OCR_00685] Johnston W. W. (1985). The malignant pleural effusion. A review of cytopathologic diagnoses of 584 specimens from 472 consecutive patients.. Cancer.

[OCR_00690] Joseph M. G., Banerjee D., Harris P., Gibson S., McFadden R. G. (1995). Multiparameter flow cytometric DNA analysis of effusions: a prospective study of 36 cases compared with routine cytology and immunohistochemistry.. Mod Pathol.

[OCR_00671] Kibbelaar R. E., Kok F., Dreef E. J., Kleiverda J. K., Cornelisse C. J., Raap A. K., Kluin P. M. (1993). Statistical methods in interphase cytogenetics: an experimental approach.. Cytometry.

[OCR_00681] LEUALLEN E. C., CARR D. T. (1955). Pleural effusion; a statistical study of 436 patients.. N Engl J Med.

[OCR_00701] Osinaga E., Pancino G., Beuzelin M., Babino A., Rodriguez D., Robello C., Tiscornia A., Phillips E., Bourguignat A., Roseto A. (1992). Detection of a soluble antigen defined by monoclonal antibody 83D4 in serous effusions associated with breast carcinoma.. Cancer.

[OCR_00725] Starr R. L., Sherman M. E. (1991). The value of multiple preparations in the diagnosis of malignant pleural effusions. A cost-benefit analysis.. Acta Cytol.

[OCR_00730] Tanner M. M., Tirkkonen M., Kallioniemi A., Holli K., Collins C., Kowbel D., Gray J. W., Kallioniemi O. P., Isola J. (1995). Amplification of chromosomal region 20q13 in invasive breast cancer: prognostic implications.. Clin Cancer Res.

[OCR_00737] Taylor C., Patel K., Jones T., Kiely F., De Stavola B. L., Sheer D. (1993). Diagnosis of Ewing's sarcoma and peripheral neuroectodermal tumour based on the detection of t(11;22) using fluorescence in situ hybridisation.. Br J Cancer.

[OCR_00744] Teixeira M. R., Pandis N., Bardi G., Andersen J. A., Mandahl N., Mitelman F., Heim S. (1994). Cytogenetic analysis of multifocal breast carcinomas: detection of karyotypically unrelated clones as well as clonal similarities between tumour foci.. Br J Cancer.

[OCR_00751] Trent J., Yang J. M., Emerson J., Dalton W., McGee D., Massey K., Thompson F., Villar H. (1993). Clonal chromosome abnormalities in human breast carcinomas. II. Thirty-four cases with metastatic disease.. Genes Chromosomes Cancer.

